# Minimal Residual Disease Detection by Next-Generation Sequencing in Multiple Myeloma: A Comparison With Real-Time Quantitative PCR

**DOI:** 10.3389/fonc.2020.611021

**Published:** 2021-01-29

**Authors:** Qiumei Yao, Yinlei Bai, Shaji Kumar, Elaine Au, Alberto Orfao, Chor Sang Chim

**Affiliations:** ^1^Department of Medicine, Queen Mary Hospital, The University of Hong Kong, Pokfulam, Hong Kong; ^2^Institute for Immunology and School of Medicine, Tsinghua University, Beijing, China; ^3^Division of Hematology, Mayo Clinic, Rochester, MN, United States; ^4^Department of Pathology, Queen Mary Hospital, The University of Hong Kong, Pokfulam, Hong Kong; ^5^Department of Medicine and Cytometry Service (Nucleus), Cancer Research Centre (IBMCC, USAL-CSIC) and CIBERONC, Institute for Biomedical Research of Salamanca (IBSAL), University of Salamanca (USAL), Salamanca, Spain

**Keywords:** minimal residual disease, multiple myeloma, next-generation sequencing, allele-specific oligonucleotide real-time quantitative-PCR, sensitivity

## Abstract

Here we compared clonotype identification by allele-specific oligonucleotide real-time quantitative-PCR (ASO RQ-PCR) and next-generation sequencing (NGS) in 80 multiple myeloma patients. ASO RQ-PCR was applicable in 49/55 (89%) and NGS in 62/78 (80%). Clonotypes identified by both methods were identical in 33/35 (94%). Sensitivity of 10^−5^ was confirmed in 28/29 (96%) by NGS while sensitivity of RQ-PCR was 10^−5^ in 7 (24%), 5 × 10^−5^ in 15 (52%), and 10^−4^ in 7 (24%). Among 14 samples quantifiable by ASO RQ-PCR, NGS yielded comparable results in 12 (86%). Applicability of NGS can be improved if immunoglobulin heavy-chain incomplete DJ primers are included.

## Introduction

Allele-specific oligonucleotide real-time quantitative-PCR (ASO RQ-PCR)-based minimal residual disease (MRD) detection is a well-established approach for treatment monitoring in multiple myeloma (MM) (1, 2). However, MM arises from post-germinal center plasma cells, in which hypermutation for enhanced antigen affinity has occurred during antigen-specific B cell responses in the germinal center of lymphoid tissues. This renders a relatively low applicability when consensus primers/probes are used for MRD detection by ASO RQ-PCR. Recently we have shown that the use of all patient-specific primers to the V and J genes in combination with a Taqman probe for complementarity-determining region 3 (CDR3) nucleotide sequences, increases the applicability of ASO RQ-PCR to 90% of patients (3, 4). Moreover, this RQ-PCR-based approach could reach a sensitivity of down to 10^−5^ by using triplicates of 500 ng DNA for MRD assessment. However, the need of patient-specific primers/probes remains labor-intensive and time-consuming and restricts its more extended use. Because of these limitations, more recently we reported on a standardized next-generation sequencing (NGS)-based protocol for MRD testing with a (uniform) sensitivity of 10^−5^ based on the use of triplicates of 1 μg DNA input and 1 million sequencing reads measured by the LymphoTrack-MiSeq platform (5, 6). Here, we compared MRD detection by both ASO RQ-PCR and NGS approaches in a series of 80 MM patients, namely their applicability (rate of successful identification of clonotypes), sensitivity and rate of quantifiable MRD cases.

## Materials and Methods

### Clonality Detection

A total of 80 MM patients were investigated for clonality at diagnosis. Among them, clonality of 25 patients was studied by NGS, 2 by ASO RQ-PCR, and 53 by both NGS and ASO RQ-PCR (**Figure S1**). In the NGS approach, clonality of diagnostic bone marrow (BM) samples was studied by four PCR assays e.g. LymphoTrack immunoglobulin heavy-chain locus (*IGH*) complete VDJ, FR1/FR2/FR3 and immunoglobulin κ locus (*IGK*) rearrangements which included both VJ and V-Kappa deleting element (Kdel) rearrangements (5). A clonal rearrangement was defined when an identical sequence with a frequency of >2.5% in a PCR amplicon based on >100,000 total sequencing reads was detected (7). In turn, for the ASO RQ-PCR approach, clonality was identified by sequential PCR of *IGH* complete VDJ FR1/FR2/FR3, *IGH* incomplete DJ, and *IGK* VJ rearrangements, followed by Sanger sequencing (3). This study was approved by the Institutional Review Board of the University of Hong Kong/Hospital Authority Hong Kong West Cluster (UW 16-111) with informed consents.

### Minimal Residual Disease Measurements by Next-Generation Sequencing and Allele-Specific Oligonucleotide Real-Time Quantitative-PCR

MRD assessment by NGS was performed as described previously (5, 6). Briefly, for each follow-up sample, triplicate measurements of 1 μg DNA input and 1 million sequencing reads were performed using a single LymphoTrack *IGH* FR1 or FR2 or FR3 or *IGK* assay according to the clonotype identified at diagnosis. In order to calculate an amplification factor two spike-in controls generated from plasmids or gDNA of purified CD138+ plasma cells of MM patients were added to each replicate, one at a concentration of 10^−5^, and the other at 10^−4^. After MRD is normalized for each replicate, final MRD levels in individual samples were defined as the mean MRD level of the corresponding triplicates. In contrast, MRD assessment by ASO RQ-PCR, required the design of ASO primers or patient-specific primers and/or probes (3). RQ-PCR was performed and interpreted according to the EuroMRD guidelines (1).

## Results and Discussion

### Assessment of Clonality in Diagnostic Samples

As a result, clonal rearrangements were identified in 89% (49/55) MM by ASO RQ-PCR and 80% (62/78) by NGS (**Figure S1** and **Table S1**). Of the six samples in which no clonal sequence was detected by Sanger sequencing, NGS did not identify any clonal sequence as well. In contrast, there were 16 other patients in whom detection of clonality by NGS failed. Among those 14 out of these 16 patients in whom Sanger sequencing performed in parallel to NGS, clonality was not detected by either method in 6/14 and 8/14 had clonality detected by Sanger sequencing (seven by *IGH* incomplete DJ and one by *IGK* VJ) (**Table S2**). Therefore, the ASO RQ-PCR approach showed superior applicability for the identification of clonality identification (89 *vs.* 80% for NGS) *via* the use of additional incomplete *IGH* DJ primer sets. By using the complete *IGH* VDJ FR1/FR2/FR3, incomplete *IGH* DJ, and *IGK* VJ rearrangements for identification of clonality, the Sequenta/Adaptive NGS platform (San Francisco, CA, USA) reported a better applicability of 91% (8). However, the applicability of our platform based on LymphoTrack-MiSeq will potentially reach similar levels of applicability if *IGH* incomplete DJ primers are also included in the assay for clonotype identification.

Thirty-five clonotypes from 34 diagnostic BM samples (patient 1 showed two distinct clonal *IGH* VDJ rearrangements) were identified by both Sanger sequencing and NGS (**Table S1**). Of the 35 clonotypes, 33 (94%) were identical. In the other two cases, while clonotypes were identified by both Sanger sequencing & NGS, the sequences were different. In patient 14, a possible reason for the discordant clonal *IGH* VDJ rearrangements by Sanger sequencing and NGS was that the myeloma plasma cells from this patient had two clonal rearrangements, whereby Sanger sequencing detected one and NGS detected the other, hence leading to two unrelated clonotypes. In case this patient will relapse in the future, investigation of the clonotypes at relapse may provide insight on the mechanism underpinning the current discordance. In turn, in patient 46, the clonal sequence identified by Sanger sequencing was an *IGK* VJ rearrangement while that of NGS was a V-Kdel rearrangement. Since primer sets for V-Kdel rearrangement were not included in the PCR followed by Sanger sequencing approach, a possible explanation for the observed discrepancy is that the clonal *IGK* VJ rearrangement was not identified by NGS. In patients 25, 43, and 44, NGS identified a second clonal sequence apart from the one identified by Sanger sequencing. Of these three patients with two clonal *IGH* or *IGK* sequences detected by NGS, one of the two clonal sequences was predicted to be functional and the other non-functional. Subsequent MRD study in patients 43 and 44 yielded concordant MRD positivity/negativity by both clonal sequences. However, the levels of MRD were different by a factor of 2.3 (0.009 *vs.* 0.004%) and 4.1 (0.049 *vs.* 0.012%), respectively, possibly due to differences in the efficiency of the PCR assays inherent with the occurrence of a mismatch at the primer binding nucleotide sequences. Whether an additional clonotype impacts MRD detection in follow-up samples requires further investigations. Similarly, Ladetto et al. compared the clonotypes of six MM patients identified by Sanger sequencing and NGS, and reported entirely unrelated *IGH* VDJ rearrangement clonotypic sequences in two patients (9). In one, the clonotype identified by Sanger sequencing was dismissed as the frequency of this clonotype was much lower than the percentage of plasma cells inside the bone marrow. In the second case, the authors could not find an explanation for the discrepancy, since no technical issue was found (9). Overall, clonotypes identified by Sanger sequencing and NGS using the same PCR are concordant. Herein, of the 35 clonotypes detected by both Sanger sequencing and NGS, only one case showed a different clonotypic sequence.

### Sensitivity of Minimal Residual Disease Detection by Next-Generation Sequencing and Allele-Specific Oligonucleotide Real-Time Quantitative-PCR

**Figure 1:**
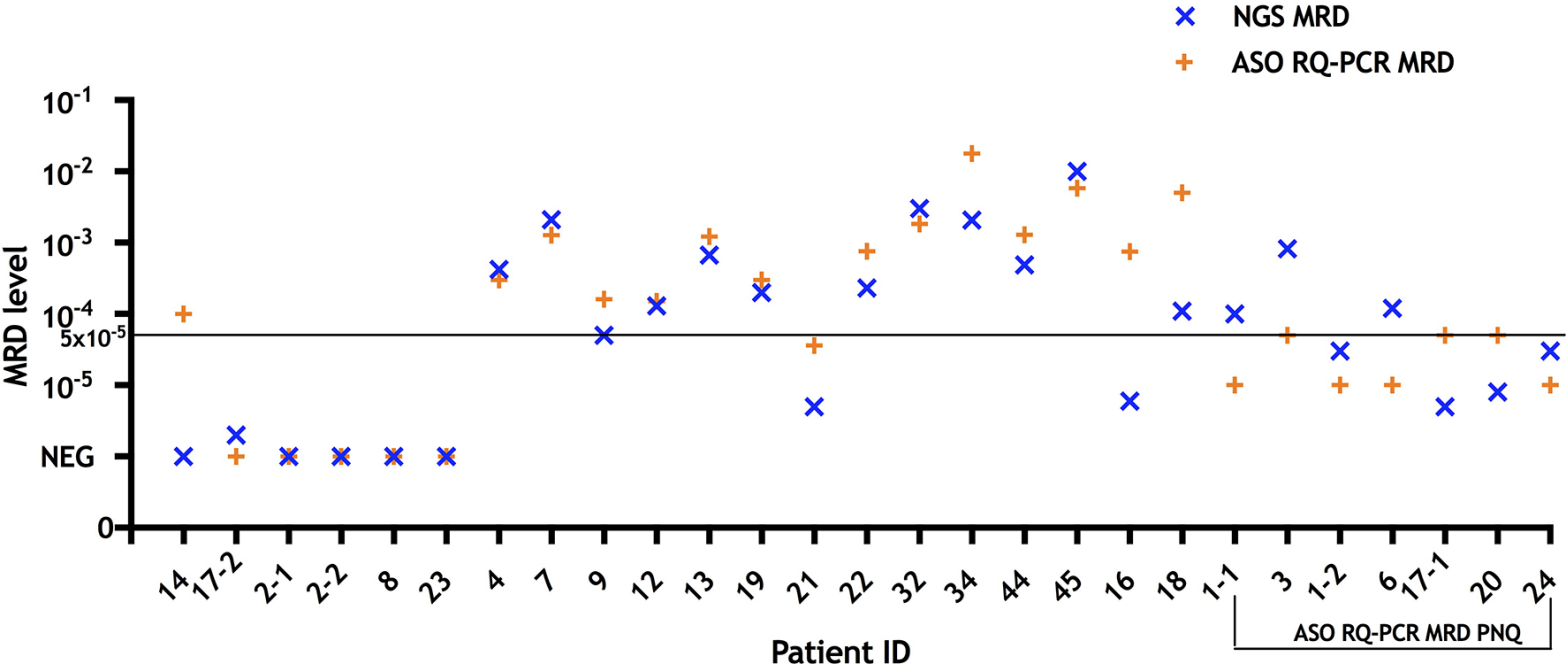
MRD results measured by NGS and ASO RQ-PCR in follow-up bone marrow samples (n = 27) from treated multiple myeloma patients (n = 24). For comparison purposes, cases that were defined as “positive not quantifiable” by ASO RQ-PCR were placed to quantitative range for that patient (10^−5^ for patients 1, 6, and 24; 5 × 10^−5^ for patients 3, 17, and 20). NGS yielded MRD+ or MRD− results concordant with ASO RQ-PCR in 25/27 samples. In patient 14 ASO RQ-PCR yielded a positive result that was not confirmed by NGS, while in one sample of patient 17 MRD was detected by NGS but not RQ-PCR with a sensitivity of 5 × 10^−5^ for that patient. Among 14 samples (patient ID 4, 7, 9, 12, 13, 19, 21, 22, 32, 34, 44, 45, 16, and 18) with quantifiable MRD by RQ-PCR, NGS yielded comparable results (differences of less than one log) in 12/14. In two samples (patients 16 and 18), MRD assessed by NGS were 2.1 and 1.7 log lower than those of RQ-PCR, respectively. MRD, minimal residual disease; NGS, next generation sequencing; ASO RQ-PCR, allele-specific oligonucleotide real-time quantitative-PCR; PNQ, positive not quantifiable.

Sensitivity of MRD detection by NGS and ASO RQ-PCR was directly compared for 29 clonotypes derived from 28 patients (**Table 1**). A sensitivity of 10^−5^ was detected in 28/29 (96%) by NGS, based on detection of the spike-in control at the 10^−5^ concentration; in contrast, the sensitivity of ASO RQ-PCR for MRD detection was of 10^−5^ in only 7/29 (24%) cases, while lower in the other patient samples: 5 × 10^−5^ in 15 (52%), and 10^−4^ in 7 (24%). Therefore, NGS showed a slightly improved sensitivity than ASO RQ-PCR for MRD detection, at the same time it obviated the need of ASO primers or patient-specific primers and/or probes. Meanwhile, in our NGS approach, sensitivity of 10^−5^ was marked by only one spike-in control, thus there was a possibility that amplification performance of the MRD target may not be reflected by the amplificability of the single rearrangement used as spike-in control. To overcome this shortage, a mix of rearrangements covering each primer set would be of value (10, 11). In a subsequent comparison of MRD results as assessed by these two approaches in 27 follow-up MM BM samples, we found only one sample (patient ID 17) in which MRD was detected by NGS but not by ASO RQ-PCR with a sensitivity of 5 × 10^−5^ for that patient (**Figure 1** and **Table S3**). In contrast, in another follow-up BM from patient 14, MRD was positive by ASO RQ-PCR with a sensitivity of 5 × 10^−5^, while not detected by NGS with a sensitivity of 10^−5^. A similar discordance has also been reported by Ladetto et al. (9) who found 8/45 (18%) follow-up samples that tested positive for MRD by ASO RQ-PCR but negative by NGS (9). In the study of Ladetto et al., the possibility of a difference in the sensitivity of the two techniques used could not be ruled out, since sensitivity of NGS was not verified in each and every follow-up sample. Another possible explanation for the observed discrepancies is the occurrence of non-specific amplification in the ASO RQ-PCR assay, which has been reported previously (12). However, in our case (patient 14), the differences observed between NGS and ASO RQ-PCR might possibly be related to the different clonal sequences identified in the diagnostic BM sample of this patient by the two techniques.

**Table 1:** Sensitivity of minimal residual disease (MRD) detection by next-generation sequencing (NGS) and allele-specific oligonucleotide real-time quantitative-PCR (ASO RQ-PCR).

Patient ID	ASO clonotypes	NGS clonotypes		Identical	NGS sensitivity	ASO RQ-PCR sensitivity
1	V_H_3.9(0)-1-7-(2)D5.12(4)-2-J_H_6	IGHV3-9_01	IGHJ6_03	Yes	10^−5^	10^−5^
	V_H_3.13(10)-5-(7)D3.22(10)-11-(1)J_H_3	IGHV3-13_01	IGHJ3_02	Yes	10^−5^	5 × 10^−5^
2	V_H_2.5(2)-6-(2)D1.26(2)-3-(5)J_H_4	IGHV2-5_09	IGHJ4_02	Yes	10^−5^	5 × 10^−5^
3	V_H_3.66(0)-5-(15)D3.16(7)-7-(14)J_H_6	IGHV3-66_02	IGHJ6_02	Yes	10^−5^	5 × 10^−5^
4	V_H_3.21(1)-9-(8)D2.21(3)-3-(5)J_H_6	IGHV3-21_02	IGHJ6_03	Yes	10^−5^	10^−4^
6	V_H_3.11(1)-15-(5)D2.21(12)-8-(10)J_H_6	IGHV3-11_05	IGHJ6_03	Yes	10^−5^	10^−5^
7	V_H_3.20(0)-10-(5)D1.26(3)-8-(4)J_H_4	IGHV3-20_01	IGHJ4_02	Yes	10^−5^	5 × 10^−5^
8	V_H_1.24(2)-(7)D1.1(1)-12-(17)J_H_5	IGHV1-24_01	IGHJ5_02	Yes	10^−5^	10^−4^
9	V_H_4.61(2)-4-(6)D2.2(12)-20-(8)J_H_3	IGHV4-61_02	IGHJ3_02	Yes	10^−5^	5 × 10^−5^
12	V_H_2.5(2)-15-(0)D5.18(3)-2-(11)J_H_4	IGHV2-5_08	IGHJ4_02	Yes	10^−5^	5 × 10^−5^
13	V_H_1.18(0)-1-6-(2)D5.24(2)-13-(19)J_H_6	IGHV1-18_01	IGHJ6_03	Yes	10^−5^	10^−5^
14	V_H_4.34(3)-14-(16)D2.2(2)-25-(16)J_H_4	IGHV3-33_01	IGHJ3_02	No	10^−5^	5 × 10^−5^
16	V_H_1.2(2)-11-(6)D4.11(2)-5-1-(0)J_H_5	IGHV1-2_02	IGHJ5_02	Yes	10^−5^	10^−4^
17	V_H_4.61(2)-4-(1)D4.23(2)-1-(8)J_H_4	IGHV4-61_01	IGHJ4_02	Yes	10^−5^	5 × 10^−5^
18	V_H_1.3(2)-8-(0)D6.19(0)-3-(7)J_H_5	IGHV1-3_01	IGHJ5_02	Yes	10^−5^	5 × 10^−5^
19	V_H_4.61(4)-8-4-D5.18(7)-11-(6)J_H_4	IGHV4-61_03	IGHJ4_02	Yes	10^−5^	5 × 10^−5^
20	V_H_3.30(3)-3-(0)D3.10(9)-4-(3)J_H_4	IGHV3-30_04	IGHJ4_02	Yes	10^−5^	5 × 10^−5^
21	V_H_4.4(2)-13-(7)D6.6(0)-3-(1)J_H_3	IGHV4-4_02	IGHJ3_01	Yes	10^−5^	10^−5^
22	V_H_4.39(0)-11-(2)D3.22(9)-8-(10)J_H_4	IGHV4-39_07	IGHJ4_02	Yes	10^−5^	10^−5^
23	V_H_5.51(3)-12-(4)D3.10(9)-4-(8)J_H_4	IGHV5-51_03	IGHJ4_02	Yes	10^−5^	10^−4^
24	V_H_3.23(0)-2-(7)D3.3(12)-0-(5)J_H_3	IGHV3-23_04	IGHJ3_01	Yes	10^−5^	10^−5^
29	V_H_3.30(0)-4-(8)D5.12(9)-4-(6)J_H_4	IGHV3-30_04	IGHJ4_02	Yes	10^−5^	10^−4^
30	V_H_2.70(5)-9-(12)D3.16(14)-3-(15)J_H_6	IGHV2-70_12	IGHJ6_02	Yes	10^−4^	5 × 10^−5^
31	D6.25(0)-5-(10)J_H_4b	IGKV1-5_03	IGKJ2_01	No	10^−5^	5 × 10^−5^
32	D4.17(3)-6-(0)J_H_4b	IGKV1-5_03	IGKJ1_01	No	10^−5^	5 × 10^−5^
34	D6.25(3)-18-(11)J_H_4b	IGKV2-29_02	IGKJ4_01	No	10^−5^	10^−4^
43	V_K_1.39(1)-2-(8)J_K_1	IGKV1D-39_01	IGKJ1_01	Yes	10^−5^	10^−4^
44	V_K_1.39(0)-0-(0)J_K_5	IGKV1D-39_01	IGKJ5_01	Yes	10^−5^	5 × 10^−5^
45	V_K_1.39(4)-0-(1)J_K_1	IGKV1D-39_01	IGKJ1_01	Yes	10^−5^	10^−5^

### Minimal Residual Disease Quantification by Next-Generation Sequencing and Allele-Specific Oligonucleotide Real-Time Quantitative-PCR

Overall, NGS and ASO RQ-PCR showed concordant positivity/negativity MRD results in the other 25/27 follow-up BM samples (positive in 21 and negative in four concordant samples) (**Figure S2**). Among those with detectable MRD, 14/21 (67%) samples were quantifiable by ASO RQ-PCR and 21/21 (100%) by NGS. Regarding accuracy of MRD quantification, ASO RQ-PCR has the advantage over NGS that the standard curve applied for quantification is constructed based on an identical clonotype sequence to that of each individual myeloma patient evaluated, while for NGS consensus spike-in controls derived from sequences other than a patient-specific one, are used. Nevertheless, our NGS platform yielded comparable MRD levels (differences of less than one log) in 12/14 (86%) to ASO RQ-PCR (**Figure 1**). In two samples (patients 16 and 18), MRD levels by NGS were 2.1 and 1.7 logs lower than those of ASO RQ-PCR, respectively. In turn, NGS provided quantitation in all those seven cases assigned “positive but not quantifiable” by ASO RQ-PCR.

In summary, ASO RQ-PCR showed superior applicability to our currently NGS approach which might still be improved if *IGH* incomplete DJ primers are incorporated in the analysis. In turn, despite a high concordance was observed as regards the specific clonotypes identified by both Sanger sequencing and NGS, NGS slightly improved sensitivity and MRD quantification, while obviating the need to design ASO primers or patient-specific primers/probes and the construction of a standard curve.

## Data Availability Statement

The original contributions presented in the study are included in the article/**Supplementary Material**; further inquiries can be directed to the corresponding author.

## Ethics Statement

The studies involving human participants were reviewed and approved by the Institutional Review Board of the University of Hong Kong/Hospital Authority Hong Kong West Cluster. The patients/participants provided their written informed consent to participate in this study.

## Author Contributions

CC designed and supervised the study. QY and YB performed the experiments. QY, YB, SK, EA, AO, and CC analyzed the data, wrote the manuscript, and approved the final version. All authors contributed to the article and approved the submitted version.

## Funding

This work was supported by the Hong Kong Blood Cancer Foundation and National Natural Science Foundation of China (81470369) awarded to CC.

## Conflict of Interest

The authors declare that the research was conducted in the absence of any commercial or financial relationships that could be construed as a potential conflict of interest.

## Supplementary Material

Supplementary Figure 1 Schematic diagram of clonality detection at diagnosis, sensitivity study, and MRD detection at follow-up, by NGS and Sanger sequencing.

Supplementary Figure 2 MRD measured by NGS and ASO RQ-PCR in 27 follow-up bone marrow samples from 24 MM patients.

Supplementary Table 1 Results of clonality identification by Sanger sequencing and next-generation sequencing.

Supplementary Table 2 Results of clonality detection by Sanger sequencing in 16 patients who failed clonality identification by next-generation sequencing.

Supplementary Table 3 Minimal residual disease levels measured by next-generation sequencing and allele-specific oligonucleotide real-time quantitative-PCR in follow-up bone marrow samples.

## Abbreviations

NGS, next generation sequencing; MRD, minimal residual disease; MM, multiple myeloma; CDR3, complementarity-determining region; ASO RQ-PCR, allele-specific oligonucleotide real-time quantitative-PCR; *IGH*, immunoglobulin heavy-chain locus; *IGK*, immunoglobulin κ locus; Kdel, Kappa deleting element; BM, bone marrow.
