# Supplementary material for: Minimal Residual Disease Detection by Next-Generation Sequencing in Multiple Myeloma: A Comparison With Real-Time Quantitative PCR
Source: Front Oncol. 2021 Jan 29;10:611021. doi: 10.3389/fonc.2020.611021 (PMC7878533; doi:10.3389/fonc.2020.611021)
Supplement: Supplementary Figure 1 — Schematic diagram of clonality detection at diagnosis, sensitivity study, and MRD detection at follow-up, by NGS and Sanger sequencing. [file DataSheet_1.docx]

**Minimal residual disease detection by next-generation sequencing in multiple myeloma: A comparison with real-time quantitative PCR**

Running title: Residual disease detection in myeloma

Qiumei Yao^1#^, Yinlei Bai^2#^, Shaji Kuma^3^, Elaine AU^4^, Alberto Orfao^5^, Chor Sang Chim1*

^1^Department of Medicine, Queen Mary Hospital, The University of Hong Kong, Pokfulam, Hong Kong

^2^Institute for Immunology and School of Medicine, Tsinghua University, Beijing, China

^3^ Division of Hematology, Mayo Clinic, Rochester, MN, USA

^4^Department of Pathology, Queen Mary Hospital, The University of Hong Kong, Pokfulam, Hong Kong

^5^Department of Medicine and Cytometry Service (Nucleus), Cancer Research Centre (IBMCC, USAL-CSIC) and CIBERONC, Institute for Biomedical Research of Salamanca (IBSAL), University of Salamanca (USAL), Salamanca, Spain

#These authors contributed equally.

*Corresponding author: Prof. Chor Sang CHIM, MD, PhD, Department of Medicine, Queen Mary Hospital, The University of Hong Kong, Pokfulam Road, Pokfulam, Hong Kong

Email: jcschim@hku.hk

Tel: (852) 2255 4769

Fax: (852) 2816 2187

**Table S1. Results of clonality identification by Sanger sequencing and next-generation sequencing.**

| Patient ID | Clonality detected by Sanger sequencing | | |  | Clonality detected by NGS | | | Type of clonal sequence |
| --- | --- | --- | --- | --- | --- | --- | --- | --- |
|  | Clonal sequence | | PCR |  | V-gene | J-gene | CDR3 identical to Sanger sequencing |  |
| Clonality done by both Sanger sequencing and NGS | |  |  |  |  |  |  |  |
| 1 | V_H_3.9(0)-1-7-(2)D5.12(4)-2-J_H_6 | | IGH VDJ |  | IGHV3-9_01 | IGHJ6_03 | Yes | Productive |
|  | V_H_3.13(10)-5-(7)D3.22(10)-11-(1)J_H_3 | | IGH VDJ |  | IGHV3-13_01 | IGHJ3_02 | Yes | Unproductive |
| 2 | V_H_2.5(2)-6-(2)D1.26(2)-3-(5)J_H_4 | | IGH VDJ |  | IGHV2-5_09 | IGHJ4_02 | Yes | Productive |
| 3 | V_H_3.66(0)-5-(15)D3.16(7)-7-(14)J_H_6 | | IGH VDJ |  | IGHV3-66_02 | IGHJ6_02 | Yes | Productive |
| 4 | V_H_3.21(1)-9-(8)D2.21(3)-3-(5)J_H_6 | | IGH VDJ |  | IGHV3-21_02 | IGHJ6_03 | Yes | Productive |
| 5 | V_H_1.18(4)-9-(1)D3.10(10)-20-(3)J_H_4 | | IGH VDJ |  | IGHV1-69_02 | IGHJ4_02 | Yes | Productive |
| 6 | V_H_3.11(1)-15-(5)D2.21(12)-8-(10)J_H_6 | | IGH VDJ |  | IGHV3-11_05 | IGHJ6_03 | Yes | Productive |
| 7 | V_H_3.20(0)-10-(5)D1.26(3)-8-(4)J_H_4 | | IGH VDJ |  | IGHV3-20_01 | IGHJ4_02 | Yes | Productive |
| 8 | V_H_1.24(2)-(7)D1.1(1)-12-(17)J_H_5 | | IGH VDJ |  | IGHV1-24_01 | IGHJ5_02 | Yes | Productive |
| 9 | V_H_4.61(2)-4-(6)D2.2(12)-20-(8)J_H_3 | | IGH VDJ |  | IGHV4-61_02 | IGHJ3_02 | Yes | Productive |
| 10 | V_H_3.66(0)-0-(0)D3.3(21)-12-(3)J_H_5 | | IGH VDJ |  | IGHV3-66_02 | IGHJ5_02 | Yes | Productive |
| 11 | V_H_3.23(5)-10-(7)D4.23(0)-5-(8)J_H_5 | | IGH VDJ |  | IGHV3-23_04 | IGHJ5_02 | Yes | Productive |
| 12 | V_H_2.5(2)-15-(0)D5.18(3)-2-(11)J_H_4 | | IGH VDJ |  | IGHV2-5_08 | IGHJ4_02 | Yes | Productive |
| 13 | V_H_1.18(0)-1-6-(2)D5.24(2)-13-(19)J_H_6 | | IGH VDJ |  | IGHV1-18_01 | IGHJ6_03 | Yes | Productive |
| 14 | V_H_4.34(3)-14-(16)D2.2(2)-25-(16)J_H_4 | | IGH VDJ |  | IGHV3-33_01 | IGHJ3_02 | No | Unproductive (Sanger)  Productive (NGS) |
| 15 | V_H_5.10(1)-15-(3)D3.22(11)-3-(3)J_H_4 | | IGH VDJ |  | IGHV5-10_01 | IGHJ4_02 | Yes | Productive |
| 16 | V_H_1.2(2)-11-(6)D4.11(2)-5-1-(0)J_H_5 | | IGH VDJ |  | IGHV1-2_02 | IGHJ5_02 | Yes | Productive |
| 17 | V_H_4.61(2)-4-(1)D4.23(2)-1-(8)J_H_4 | | IGH VDJ |  | IGHV4-61_01 | IGHJ4_02 | Yes | Productive |
| 18 | V_H_1.3(2)-8-(0)D6.19(0)-3-(7)J_H_5 | | IGH VDJ |  | IGHV1-3_01 | IGHJ5_02 | Yes | Productive |
| 19 | V_H_4.61(4)-8-4-D5.18(7)-11-(6)J_H_4 | | IGH VDJ |  | IGHV4-61_03 | IGHJ4_02 | Yes | Productive |
| 20 | V_H_3.30(3)-3-(0)D3.10(9)-4-(3)J_H_4 | | IGH VDJ |  | IGHV3-30_04 | IGHJ4_02 | Yes | Productive |
| 21 | V_H_4.4(2)-13-(7)D6.6(0)-3-(1)J_H_3 | | IGH VDJ |  | IGHV4-4_02 | IGHJ3_01 | Yes | Productive |
| 22 | V_H_4.39(0)-11-(2)D3.22(9)-8-(10)J_H_4 | | IGH VDJ |  | IGHV4-39_07 | IGHJ4_02 | Yes | Productive |
| 23 | V_H_5.51(3)-12-(4)D3.10(9)-4-(8)J_H_4 | | IGH VDJ |  | IGHV5-51_03 | IGHJ4_02 | Yes | Productive |
| 24 | V_H_3.23(0)-2-(7)D3.3(12)-0-(5)J_H_3 | | IGH VDJ |  | IGHV3-23_04 | IGHJ3_01 | Yes | Productive |
| 25 | V_H_1.68(6)-4-(5)D6.13(2)-11-(14)J_H_6 | | IGH VDJ |  | IGHV1-68_02 | IGHJ6_02 | Yes | Unproductive |
|  |  | |  |  | IGHV3-20_01 | IGHJ4_02 |  | Productive |
| 26 | V_H_3.30(2)-7-(2)D3.3(15)-11-(8)J_H_4 | | IGH VDJ |  | IGHV3-30-3_01 | IGHJ4_02 | Yes | productive |
| 27 | V_H_3.53(2)-3-(9)D3.9(12)-5-(11)J_H_6 | | IGH VDJ |  | IGHV3-53_02 | IGHJ6_02 | Yes | Productive |
| 28 | V_H_4.39 (1)-(7)D5.12(5)-19-(12)J_H_6 | | IGH VDJ |  | IGHV4-39_07 | IGHJ6_02 | Yes | Productive |
| 29 | V_H_3.30(0)-4-(8)D5.12(9)-4-(6)J_H_4 | | IGH VDJ |  | IGHV3-30_04 | IGHJ4_02 | Yes | Productive |
| 30 | V_H_2.70(5)-9-(12)D3.16(14)-3-(15)J_H_6 | | IGH VDJ |  | IGHV2-70_12 | IGHJ6_02 | Yes | Productive |
| 31 | D6.25(0)-5-(10)J_H_4b | | IGH DJ |  | IGKV1-5_03 | IGKJ2_01 | N/A | N/A (Sanger)  Productive (NGS) |
| 32 | D4.17(3)-6-(0)J_H_4b | | IGH DJ |  | IGKV1-5_03 | IGKJ1_01 | N/A | N/A (Sanger)  Productive (NGS) |
| 33 | D2.2(11)- 12-(6)J_H_5b | | IGH DJ |  | IGKV3-11_01 | IGKJ4_01 | N/A | N/A (Sanger)  Productive (NGS) |
| 34 | D6.25(3)-18-(11)J_H_4b | | IGH DJ |  | IGKV2-29_02 | IGKJ4_01 | N/A | N/A (Sanger)  Productive (NGS) |
|  |  | |  |  | IGKV1-6_01 | IGKJ1_01 |  | N/A (Sanger)  Productive (NGS) |
| 35 | D2.2(19)-9-(6)J_H_4b | | IGH DJ |  | IGKV2D-29_01 | IGKJ2_03 | N/A | N/A (Sanger)  Unproductive (NGS) |
| 36 | D2.2(6)-6-(2)J_H_5b | | IGH DJ |  | No clonality |  |  |  |
| 37 | D6.25(3)-20-(12)J_H_4b | | IGH DJ |  | No clonality |  |  |  |
| 38 | D1.26(4)-7-(0)J_H_3b | | IGH DJ |  | No clonality |  |  |  |
| 39 | D2.2(4)-8-(15)JH6b | | IGH DJ |  | No clonality |  |  |  |
| 40 | D6.13(2)-0-(9)J_H_4b | | IGH DJ |  | No clonality |  |  |  |
| 41 | D3.9(17)-12-(2)J_H_5b | | IGH DJ |  | No clonality |  |  |  |
| 42 | D1.26(2)-11-(5)J_H_5b | | IGH DJ |  | No clonality |  |  |  |
| 43 | V_K_1.39(1)-2-(8)J_K_1 | | IGK |  | IGKV1D-39_01 | IGKJ1_01 | Yes | Unproductive |
|  |  | |  |  | IGKV1D-33_01 | IGKJ4_01 |  | Productive |
| 44 | V_K_1.39(0)-0-(0)J_K_5 | | IGK |  | IGKV1D-39_01 | IGKJ5_01 | Yes | Productive |
|  |  | |  |  | IGKV1D-33_01 | IGKJ4_02 |  | Unproductive |
| 45 | V_K_1.39(4)-0-(1)J_K_1 | | IGK |  | IGKV1D-39_01 | IGKJ1_01 | Yes | Unproductive |
| 46 | V_K_1.12(1)-0-(2)J_K_3 | | IGK |  | IGKV3D-20_01 | Kdel | No | NA (Sanger)  N/A (NGS) |
| 47 | V_K_1.5(8)-6-(2)J_K_2 | | IGK |  | no clonality |  |  | NA (Sanger) |
| 48 | No clonality | |  |  | No clonality |  |  |  |
| 49 | No clonality | |  |  | No clonality |  |  |  |
| 50 | No clonality | |  |  | No clonality |  |  |  |
| 51 | No clonality | |  |  | No clonality |  |  |  |
| 52 | No clonality | |  |  | No clonality |  |  |  |
| 53 | No clonality | |  |  | No clonality |  |  |  |
| Clonality done by Sanger sequencing only | |  |  |  |  |  |  |  |
| 54 | V_H_3.9(1)-5-(2)D6.6-(0)-13-(15)J_H_6 | | IGH VDJ |  |  |  |  | NA (Sanger) |
| 55 | D2.15(2)-15-(0)JH6 | | IGH DJ |  |  |  |  |  |
| Clonality done by NGS only | |  |  |  |  |  |  |  |
| 56 |  | |  |  | IGHV3-7_01 | IGHJ4_02 |  | Unproductive |
|  |  | |  |  | IGHV3-64_05 | IGHJ4_02 |  | Productive |
| 57 |  | |  |  | IGHV2-70_01 | IGHJ6_02 |  | Productive |
| 58 |  | |  |  | IGKV4-1_01 | Kdel |  | N/A |
| 59 |  | |  |  | IGHV3-21_02 | IGHJ4_02 |  | Productive |
| 60 |  | |  |  | IGHV3-23_04 | IGHJ6_02 |  | Productive |
| 61 |  | |  |  | IGHV3-74_01 | IGHJ4_02 |  | Productive |
| 62 |  | |  |  | IGHV4-39_01 | IGHJ5_02 |  | Productive |
| 63 |  | |  |  | IGKV2D-40_01 | IGKJ4_01 |  | Unproductive |
| 64 |  | |  |  | IGKV2D-26_01 | Kdel |  | N/A |
| 65 |  | |  |  | IGKV1D-33_01 | IGKJ2_01 |  | Productive |
| 66 |  | |  |  | IGHV4-b_02 | IGHJ4_02 |  | Productive |
| 67 |  | |  |  | IGHV3-48_01 | IGHJ6_02 |  | Productive |
| 68 |  | |  |  | IGKV2D-28_01 | Kdel |  | N/A |
| 69 |  | |  |  | IGHV3-30_07 | IGHJ1_01 |  | Productive |
| 70 |  | |  |  | IGHV2-5_10 | IGHJ4_02 |  | Productive |
|  |  | |  |  | IGHV4-30-4_06 | IGHJ6_03 |  | Unproductive |
| 71 |  | |  |  | IGHV4-59_07 | IGHJ4_02 |  | Productive |
| 72 |  | |  |  | IGHV3-48_03 | IGHJ4_02 |  | Productive |
| 73 |  | |  |  | IGHV4-39_03 | IGHJ5_02 |  | Productive |
| 74 |  | |  |  | IGHV2-26_01 | IGHJ6_02 |  | Productive |
| 75 |  | |  |  | IGHV4-59_01 | IGHJ6_02 |  | Productive |
| 76 |  | |  |  | IGHV3-74_03 | IGHJ4_02 |  | Productive |
| 77 |  | |  |  | IGKV5-2_01 | IGKJ1_01 |  | Unproductive |
| 78 |  | |  |  | IGHV6-1_02 | IGHJ5_02 |  | Productive |
| 79 |  | |  |  | No clonality |  |  |  |
| 80 |  | |  |  | No clonality |  |  |  |

Abbreviations: NGS: next-generation sequencing; Kdel: Kappa deleting element; N/A: not applicable; NA: not available; IGH VDJ: IGH complete VDJ rearrangement; IGH DJ: IGH incomplete DJ rearrangement; IGK: IGK VJ rearrangement in Sanger sequencing, IGK VJ and V-Kdel rearrangement in NGS PCR.

**Table S2. Results of clonality detection by Sanger sequencing in 16 patients who failed clonality identification by next-generation sequencing.**

| Sanger sequencing | Number of patients (n) |
| --- | --- |
| Unsuccessful | 6 |
| Successful | 8 |
| Not assessed | 2 |
| Total | 16 |

**Table S3. Minimal residual disease (MRD) levels measured by next-generation sequencing and allele-specific oligonucleotide real-time quantitative-PCR (ASO RQ-PCR) in follow-up bone marrow samples.**

| Patient ID | Replicate | Sequencing reads (million) | 10^-5^ control reads | 10^-4^ control reads (frequency) | MRD reads | Normalized MRD | Mean MRD | ASO RQ-PCR MRD |
| --- | --- | --- | --- | --- | --- | --- | --- | --- |
| 1 (FU1) | -1 | 0.8 | 0 | 13128 (1.5x10^-2^) | 1737 | 0.0013% | 0.0004% | PNQ<10 |
|  | -2 | 0.9 | 1279 | 8216 (0.9x10^-2^) | 0 | 0 |  |  |
|  | -3 | 0.5 | 858 | 9344 (2.0x10^-2^) | 0 | 0 |  |  |
| 1 (FU2) | -1 | 2.0 | 11 | 702 (3.5x10^-4^) | 1021 | 0.016% | 0.010% | PNQ<50 |
|  | -2 | 1.2 | 250 | 616 (5.2x10^-4^) | 807 | 0.013% |  |  |
|  | -3 | 1.7 | 0 | 407 (2.4x10^-4^) | 141 | 0.003% |  |  |
| 2 (FU1) | -1 | 1.2 | 0 | 65 (5.5x10^-5^) | 0 | 0 | Negative | Negative |
|  | -2 | 1.0 | 11 | 70 (5.5x10^-5^) | 0 | 0 |  |  |
|  | -3 | 0.9 | 1 | 43 (4.8x10^-5^) | 0 | 0 |  |  |
| 2 (FU2) | -1 | 1.1 | 0 | 369 (3.4x10^-4^) | 0 | 0 | Negative | Negative |
|  | -2 | 0.5 | 5 | 0 | 0 | 0 |  |  |
|  | -3 | 0.8 | 48 | 516 (6.7x10^-4^) | 0 | 0 |  |  |
| 3 | -1 | 1.4 | 185 | 208 (1.4x10^-4^) | 3547 | 0.17% | 0.082% | PNQ<50 |
|  | -2 | 2.0 | 8 | 730 (3.7x10^-4^) | 1962 | 0.027% |  |  |
|  | -3 | 1.1 | 5 | 340 (3.2x10^-4^) | 1674 | 0.049% |  |  |
| 4 | -1 | 2.9 | 99 | 111 (3.8x10^-5^) | 687 | 0.061% | 0.042% | 30 |
|  | -2 | 1.3 | 9 | 120 (9.3x10^-5^) | 280 | 0.023% |  |  |
|  | -3 | 2.1 | 7 | 5 (2.3x10^-6^) | 311 | N/A |  |  |
| 6 | -1 | 1.4 | 298 | 729 (5.1x10^-4^) | 242 | 0.003% | 0.012% | PNQ<10 |
|  | -2 | 1.8 | 0 | 612 (3.4x10^-4^) | 1790 | 0.029% |  |  |
|  | -3 | 1.4 | 856 | 1206 (8.9x10^-4^) | 562 | 0.005% |  |  |
| 7 | -1 | 1.5 | 0 | 601 (4.0x10^-4^) | 18207 | 0.303% | 0.209% | 128/10^5^cells |
|  | -2 | 1.3 | 0 | 1399 (1.1x10^-3^) | 21781 | 0.156% |  |  |
|  | -3 | 1.2 | 144 | 1243 (1.0x10^-3^) | 20949 | 0.169% |  |  |
| 8 | -1 | 1.5 | 184 | 101 (6.7x10^-5^) | 0 | 0 | Negative | Negative |
|  | -2 | 1.0 | 0 | 280 (2.9x10^-4^) | 0 | 0 |  |  |
|  | -3 | 1.2 | 83 | 154 (1.3x10^-4^) | 0 | 0 |  |  |
| 9 | -1 | 0.4 | 0 | 12123 (3.4x10^-2^) | 8757 | 0.007% | 0.005% | 16/10^5^ cells |
|  | -2 | 0.4 | 464 | 15234 (4.3x10^-2^) | 10007 | 0.007% |  |  |
|  | -3 | 0.5 | 2187 | 21140 (4.4x10^-2^) | 4541 | 0.002% |  |  |
| 12 | -1 | 1.2 | 108 | 1500 (1.4x10^-3^) | 1076 | 0.007% | 0.013% | 12/10^5^ cells |
|  | -2 | 1.0 | 100 | 569 (0.6x10^-3^) | 658 | 0.012% |  |  |
|  | -3 | 1.3 | 221 | 579 (0.5x10^-3^) | 1155 | 0.020% |  |  |
| 13 | -1 | 1.7 | 0 | 1548 (1.0x10^-3^) | 9883 | 0.064% | 0.067% | 122/10^5^ cells |
|  | -2 | 1.4 | 187 | 1462 (1.0x10^-3^) | 10616 | 0.073% |  |  |
|  | -3 | 0.8 | 17 | 934 (1.1x10^-3^) | 6294 | 0.067% |  |  |
| 14 | -1 | 1.0 | 54 | 219 (2.3x10^-4^) | 0 | 0 | Negative | PNQ<10 |
|  | -2 | 0.9 | 0 | 84 (9.2x10^-5^) | 0 | 0 |  |  |
|  | -3 | 0.9 | 188 | 149 (1.6x10^-4^) | 0 | 0 |  |  |
| 16 | -1 | 1.2 | 35 | 233 (1.9x10^-4^) | 20 | 0.0009% | 0.0006% | 75/10^5^ cells |
|  | -2 | 1.4 | 146 | 346 (2.4x10^-4^) | 18 | 0.0005% |  |  |
|  | -3 | 1.2 | 0 | 313 (2.6x10^-4^) | 13 | 0.0005% |  |  |
| 17 (FU1) | -1 | 1.3 | 377 | 2125 (1.6x10^-3^) | 64 | 0.0005% | 0.0005% | PNQ<50 |
|  | -2 | 1.3 | 72 | 2101 (1.6x10^-3^) | 82 | 0.0005% |  |  |
|  | -3 | 1.4 | 174 | 6587 (4.6x10^-3^) | 99 | 0.0005% |  |  |
| 17 (FU2) | -1 | 1.1 | 2054 | 6751 (6.1x10^-3^) | 46 | 0.0005% | 0.0003% | Negative |
|  | -2 | 1.3 | 1397 | 8120 (6.1x10^-3^) | 36 | 0.0005% |  |  |
|  | -3 | 1.2 | 1802 | 11728 (9.7x10^-3^) | 0 | 0 |  |  |
| 18 | -1 | 1.3 | 682 | 6827 (1.6x10^-3^) | 4097 | 0.008% | 0.011% | 503/10^5^ cells |
|  | -2 | 1.2 | 1224 | 1892 (1.6x10^-3^) | 4269 | 0.022% |  |  |
|  | -3 | 1.5 | 1695 | 7078 (1.6x10^-3^) | 4322 | 0.006% |  |  |
| 19 | -1 | 1.2 | 47 | 1456 (1.2x10^-3^) | 3080 | 0.02% | 0.02% | 30/10^5^ cells |
|  | -2 | 1.2 | 0 | 1129 (0.9x10^-3^) | 2506 | 0.02% |  |  |
|  | -3 | 1.3 | 0 | 1508 (1.2x10^-3^) | 2612 | 0.02% |  |  |
| 20 | -1 | 1.2 | 177 | 3066 (2.6x10^-3^) | 0 | 0 | 0.0008% | PNQ<10 |
|  | -2 | 1.6 | 405 | 7442 (4.6x10^-3^) | 870 | 0.0012% |  |  |
|  | -3 | 1.2 | 0 | 4085 (3.4x10^-3^) | 609 | 0.0015% |  |  |
| 21 | -1 | 0.9 | 118 | 1632 (1.8x10^-3^) | 5 | 0.0005% | 0.0005% | 3.6/10^5^ cells |
|  | -2 | 0.8 | 115 | 979 (1.2x10^-3^) | 52 | 0.0005% |  |  |
|  | -3 | 1.0 | 0 | 1624 (1.7x10^-3^) | 21 | 0.0005% |  |  |
| 22 | -1 | 1.4 | 0 | 19143 (1.4x10^-2^) | 44946 | 0.023% | 0.023% | 76/10^5^ cells |
|  | -2 | 1.2 | 0 | 19897 (1.6x10^-2^) | 45450 | 0.023% |  |  |
|  | -3 | 1.3 | 10111 | 25462 (1.9x10^-2^) | 61598 | 0.024% |  |  |
| 23 | -1 | 0.7 | 2255 | 6005 (8.5x10^-3^) | 0 | 0 | Negative | Negative |
|  | -2 | 1.1 | 2924 | 7490 (6.9x10^-3^) | 0 | 0 |  |  |
|  | -3 | 1.2 | 826 | 4637 (3.8x10^-3^) | 0 | 0 |  |  |
| 24 | -1 | 1.1 | 2544 | 6263 (6.0x10^-3^) | 0 | 0 | 0.0029% | PNQ<10 |
|  | -2 | 1.1 | 1563 | 3695 (3.5x10^-3^) | 0 | 0 |  |  |
|  | -3 | 0.9 | 0 | 3280 (3.6x10^-3^) | 2856 | 0.0087% |  |  |
| 29 | -1 | 1.2 | 10564 | 28172 (2.4x10^-2^) | 8882 | 0.0032% | 0.0089% | not done |
|  | -2 | 1.5 | 4 | 26516 (1.8x10^-2^) | 53399 | 0.021% |  |  |
|  | -3 | 1.3 | 6350 | 37562 (2.9x10^-2^) | 12676 | 0.0034% |  |  |
| 30 | -1 | 1.1 | 0 | 603 (5.7x10^-4^) | 3 | 0.0005% | 0.0002% | not done |
|  | -2 | 1.1 | 0 | 545 (5.1x10^-4^) | 0 | 0 |  |  |
|  | -3 | 1.5 | 0 | 581 (3.8x10^-4^) | 0 | 0 |  |  |
| 31 | -1 | 1.3 | 8 | 3608 (2.7x10^-3^) | 5732 | 0.016% | 0.021% | not done |
|  | -2 | 1.3 | 1673 | 2432 (1.9x10^-3^) | 4583 | 0.019% |  |  |
|  | -3 | 1.0 | 643 | 1750 (1.7x10^-3^) | 5122 | 0.029% |  |  |
| 32 | -1 | 1.1 | 0 | 229 (2.1x10^-4^) | 6926 | 0.3% | 0.300% | 184/10^5^ cells |
|  | -2 | 1.1 | 131 | 281 (2.6x10^-4^) | 6909 | 0.2% |  |  |
|  | -3 | 1.2 | 0 | 228 (1.9x10^-4^) | 7945 | 0.3% |  |  |
| 34 | -1 | 1.0 | 43 | 167 (1.7x10^-4^) | 2401 | 0.144% | 0.207% | 1758/10^5^cells |
|  | -2 | 1.0 | 50 | 66 (6.7x10^-5^) | 2352 | 0.357% |  |  |
|  | -3 | 0.6 | 57 | 113 (1.8x10^-4^) | 1349 | 0.119% |  |  |
| 43 | -1 | 0.9 | 48 | 79 (8.7x10^-5^) | 0 | 0 | Negative | not done |
|  | -2 | 1.1 | 41 | 157 (1.4x10^-4^) | 0 | 0 |  |  |
|  | -3 | 0.9 | 0 | 70 (7.5x10^-5^) | 0 | 0 |  |  |
| 44 | -1 | 0.9 | 5 | 1819 (2.1x10^-3^) | 9497 | 0.052% | 0.049% | 129/10^5^cells |
|  | -2 | 0.7 | 0 | 2742 (3.7x10^-3^) | 11814 | 0.043% |  |  |
|  | -3 | 1.1 | 0 | 2907 (3.8x10^-3^) | 15268 | 0.053% |  |  |
| 45 | -1 | 1.3 | 35 | 81 (6.1x10^-5^) | 9595 | 1.2% | 1% | 584/10^5^ cells |
|  | -2 | 1.7 | 102 | 114 (6.8x10^-5^) | 11574 | 1.0% |  |  |
|  | -3 | 1.4 | 0 | 128 (9.2x10^-5^) | 10030 | 0.8% |  |  |

Abbreviations: FU: follow-up; PNQ: positive but not quantifiable; N/A: not applicable.

**
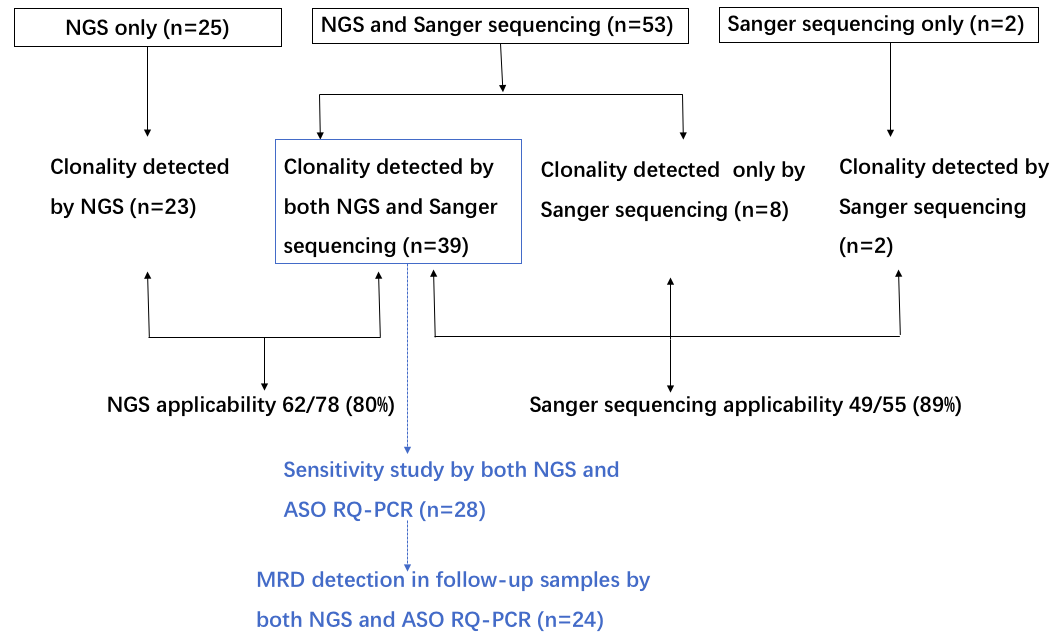
**

**Figure S1. Schematic diagram of clonality detection at diagnosis, sensitivity study and MRD detection at follow-up, by NGS and Sanger sequencing. NGS: next-generation sequencing; ASO RQ-PCR: allele-specific oligonucleotide real-time quantitative-PCR; MRD: minimal residual disease.**


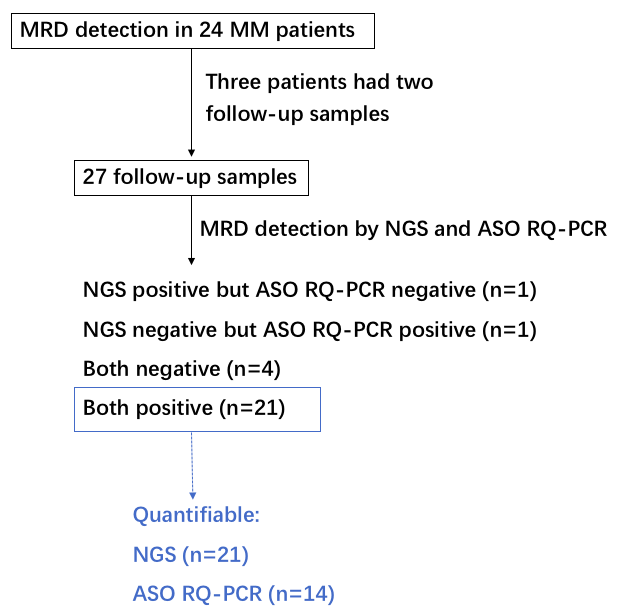


**Figure S2. MRD measured by NGS and ASO RQ-PCR in 27 follow-up bone marrow samples from 24 MM patients. MM: multiple myeloma; NGS: next-generation sequencing; ASO RQ-PCR: allele-specific oligonucleotide real-time quantitative-PCR; MRD: minimal residual disease.**
